# Editorial: Eosinophilic inflammation in chronic lung diseases: emerging molecular insights and therapeutic strategy

**DOI:** 10.3389/fmed.2025.1698140

**Published:** 2025-10-17

**Authors:** Wun-Hao Cheng, Kian Fan Chung, Ting-Yu Lin, Hsiao-Chi Chuang

**Affiliations:** ^1^School of Respiratory Therapy, College of Medicine, Taipei Medical University, Taipei, Taiwan; ^2^National Lung & Heart Institute, Imperial College London, London, United Kingdom; ^3^Department of Thoracic Medicine, Chang Gung Memorial Hospital at Linkou, Taoyuan, Taiwan; ^4^College of Medicine, Chang Gung University, Taoyuan, Taiwan; ^5^Division of Pulmonary Medicine, Department of Internal Medicine, Shuang Ho Hospital, Taipei Medical University, Taipei, Taiwan; ^6^Cell Physiology and Molecular Image Research Center, Wan Fang Hospital, Taipei Medical University, Taipei, Taiwan

**Keywords:** eosinophil, inflammation, chronic obstructive pulmonary disease (COPD), climate change, epithelia cells

Eosinophils have shifted from being single-purpose effectors to multipurpose regulators with distinct subtypes and context-dependent roles across many conditions such as asthma, COPD, and eosinophilic granulomatosis with polyangiitis (EGPA). This Research Topic provides new conceptual and translational advances that connect resident vs. inflammatory eosinophil states and their potential biomarkers (Sanchez Santos et al.), mitochondrial control of eosinophil survival, activation and extracellular trap formation (Koranteng et al.), IL-18/inflammasome pathways that sustain severe, treatment-refractory airway inflammation (Thawanaphong et al.), real-world performance of IL-5/IL-5R biologics in EGPA-asthma (Desaintjean et al.), and climate-linked extreme temperatures as upstream modulators of epithelial gene programs (Makrufardi et al.). Together, these publications argue for a shift from relying solely on blood eosinophil counts toward integrated endotyping that combines airway compartment biomarkers (e.g., EPX and IL-18), environmental exposures, and cell-intrinsic metabolic cues to guide therapy. We highlight the key convergences in this the issue that focuses on standardized eosinophil subtyping, comprehensive airway compartment biomarker panels, mitochondria-targeted adjuvants, IL-18, and inflammasome modulation, and climate-responsive clinical pathways.

Eosinophil heterogeneity is being increasingly recognized, particularly the distinction between resident (rEos) and inflammatory eosinophils (iEos). These subsets, with distinct surface phenotypes and tissue niches, change how we interpret blood counts, clarify discrepancies between peripheral and tissue eosinophilia, and point toward subpopulation-specific treatment strategies (Sanchez Santos et al.). Whether these represent true lineages or activation states remains an open question, and their dynamic shifts during exacerbations in asthma and COPD add another layer of complexity. Understanding these subsets may help explain the variable responses to biologics targeting eosinophils and refine endotyping strategies that move beyond a simple numeric threshold.

Mitochondria have emerged as critical rheostats for eosinophil biology. Their control extends to intrinsic apoptosis, survival, activation, metabolic plasticity, and extracellular trap formation. Pro-survival cytokines such as IL-5 and GM-CSF amplify oxidative phosphorylation and glycolysis, while corticosteroids accelerate apoptosis. These metabolic programs intersect with mitochondrial alterations in airway epithelial and smooth muscle cells, suggesting shared stress-response pathways across cell types (Koranteng et al.). This raises the intriguing possibility that mitochondrial modulators, whether enhancing mitophagy or tuning reactive oxygen species, could act as adjuvants layered onto anti-T2 biologics in severe asthma. To achieve this, harmonized assays for eosinophil bioenergetics and extracellular trap formation, paired with airway biomarkers such as sputum EPX and IL-18, will be essential in longitudinal studies.

The IL-18/inflammasome axis has gained prominence as a driver of residual disease activity in patients inadequately controlled on IL-5 or IL-5R biologics. Elevated sputum IL-18 correlates with incomplete response and may sustain airway hyperresponsiveness, mucus production, and remodeling through both T2 and non-T2 inflammatory programs (Thawanaphong et al.). This finding highlights the need for composite clinical endpoints that include airway IL-18 and EPX measurements when eosinophils are pharmacologically suppressed. A logical next step is biomarker-enriched trials that test IL-18 pathway modulators as add-on treatments in severe asthma patients with evidence of IL-18 signatures.

In real-world practice, retrospective cohort data demonstrate that mepolizumab and benralizumab in EGPA-asthma patients reduce steroid burden and exacerbation frequency over 12 months, though FEV1 gains remain modest. Safety profiles are acceptable, confirming guideline recommendations but also suggests the persistence of uncontrolled pathways such as IL-18 (Desaintjean et al.). EGPA-asthma thus becomes an example of the need for precision dosing and for combining IL-5 axis therapies with biomarker-guided adjuncts.

Environmental factors also shape eosinophilic airway disease, as demonstrated by research showing that extreme temperatures and rapid fluctuations alter airway epithelial gene expression in both animal models and in humans (Makrufardi et al.). This work provides compelling evidence that stressors resulting from climate change intersect with mitochondrial stress responses and inflammasome activation, further influencing eosinophil-centered pathophysiology. Incorporating exposure histories to such indices as heat and cold into the phenotyping framework may help identify vulnerable subgroups and inform climate-responsive clinical strategies, including real-time alerts and counseling during extreme weather events.

In COPD, eosinophils continue to emerge as prognostic biomarkers. A large real-world dataset indicates that higher blood eosinophil counts (≥300/μl) are associated with increased risks of exacerbations, acute respiratory failure, and mortality over 3 years (Hsu et al.). Intriguingly, blood eosinophils in COPD may harbor disproportionately low fractions of iEos, which could help explain muted responses to IL-5 axis biologics. Future COPD trials should thus include eosinophil subtyping and airway-compartment biomarker assessment, such as in the study of dupilumab in high-eosinophil COPD patients that showed a significant reduction in excaerbations through an iEos-aware framework.

In summary, the studies presented in this Research Topic call for the standardization of eosinophil subtyping, including consensus gating and definitions across blood, sputum, and tissue compartments (Sanchez Santos et al.). Adoption of airway biomarkers such as EPX and IL-18 into clinical practice could be prioritized, with well-defined cutoffs and temporal kinetics (Thawanaphong et al.). Mitochondrial modulation strategies deserve exploration in pilot add-on trials embedded with mechanistic endpoints (Koranteng et al.). Early-phase studies of IL-18 blockade and inflammasome inhibition should be conducted in biomarker-positive patients who do not respond adequately to eosinophil depletion. Finally, exposure to climate changes needs to be integrated into clinical monitoring and trial design, with weather-triggered step-up protocols tested prospectively (Makrufardi et al.).

